# Uncommon cause of trigeminal neuritis and central nervous system involvement by herpes labialis: a case report

**DOI:** 10.1186/s12883-022-02823-x

**Published:** 2022-08-05

**Authors:** Hyunsoo Kim, Kyung Wook Kang, Jae-Myung Kim, Man-Seok Park

**Affiliations:** Department of Neurology, Chonnam National University Medical School and Hospital, 42 Jebong-ro, Dong-gu, Gwangju, 58128 South Korea

**Keywords:** Trigeminal neuritis, Herpes labialis, Central nervous system vasculitis

## Abstract

**Background:**

Trigeminal neuropathy is characterized by numbness in the region innervated by the trigeminal nerves, with or without neuropathic weakness in the muscles of mastication. Trigeminal neuritis is a form of trigeminal neuropathy in which the lesion is caused by an inflammation. Herein, we report a patient with trigeminal neuritis due to central nervous system (CNS) involvement of herpes labialis (HL) infection, which was successfully treated with anti-viral and anti-inflammatory agents.

**Case presentation:**

A young healthy female presented with numbness in the left hemiface for two weeks. She had a preceding typical HL infection on left facial lip one week before the sensory symptom onset. Brain magnetic resonance imaging revealed high signal intensities and asymmetrical thickening with enhancement along the cisternal segment of the left trigeminal nerve. Additionally, brain MR angiography showed multifocal stenoses in the M1 segment of the middle cerebral artery and the cavernous portion of the internal carotid artery. Cerebrospinal fluid (CSF) examination showed mild pleocytosis with normal protein level, glucose ratio, but CSF polymerase chain reaction assay for specific anti-viral antibodies including herpes simplex virus was negative, and CSF culture also did not identify a specific pathogen. The results of serologic testing including tumor markers and autoimmune markers were all unremarkable. A tentative diagnosis of trigeminal neuritis as a complication of HL involving the CNS was made considering the clinical, neuroradiological, and laboratory findings of the patient. Therefore, the patient was treated with intravenous methylprednisolone and acyclovir for 10 days. After the treatments, her sensory disturbance was markedly improved. Brain MRI at the 3-month follow-up also demonstrated improvement of previously identified high signal intensity lesions and multifocal intracerebral artery stenoses.

**Conclusion:**

HL is usually a self-limiting, benign disease without complications, but rarely presents as trigeminal neuritis due to CNS involvement. Therefore, meticulous evaluation may be necessary if trigeminal neuritis or CNS involving symptoms occur after HL.

## Background

Trigeminal neuropathy is characterized by numbness in the region innervated by the trigeminal nerves, with or without neuropathic weakness in the muscles of mastication [1]. Trigeminal neuropathy may arise from various conditions including trauma, neoplasm, infection, and central nervous system (CNS) inflammatory disease, although the causes remained uncertain for many cases [2]. Among them, trigeminal neuritis is a form of trigeminal neuropathy in which the lesion presumably is caused by an inflammation [3]. Herein, we report a patient with trigeminal neuritis due to CNS involvement of herpes labialis (HL) infection, which was successfully treated with anti-viral and anti-inflammatory agents.

## Case presentation

**Fig. 1:**
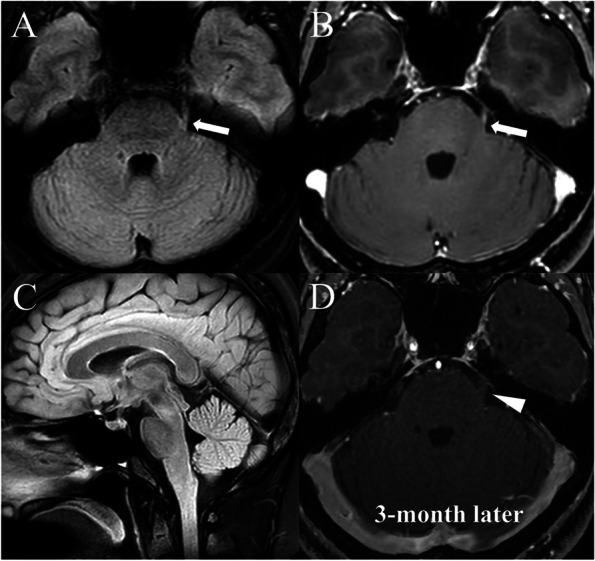
The imaging findings of trigeminal neuritis initial and after treatment. Fluid-Attenuated Inversion Recovery (FLAIR) imaging and gadolinium enhancement T1-weighted imaging shows a high signal intensity lesion in the left trigeminal nerve (arrow) suggestive of trigeminal neuritis (Fig. 1**A** and 1**B**). On sagittal FLAIR imaging, there was no evidence of other central nervous system demyelinating disease (Fig. 1**C**). The follow-up MRI, conducted 3 months later, shows an improved state of the previously observed trigeminal neuritis (arrowhead, Fig. 1**D**)

**Fig. 2:**
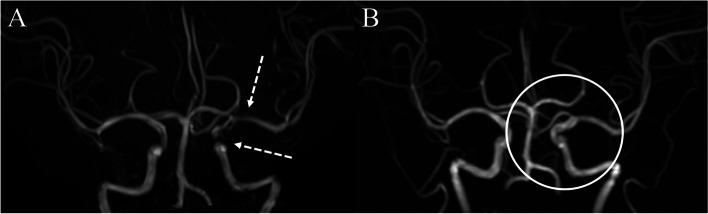
The imaging findings of multifocal cerebral arterial stenosis initial and after treatment. Initially conducted brain magnetic resonance angiography (MRA) shows multifocal arterial stenosis in the cavernous portion of the left internal carotid artery and M1 portion of the left middle cerebral artery (dotted arrow, Fig. 2**A**). The follow-up MRA, conducted 3 months later, shows an improved state of previously seen multifocal cerebral arterial stenosis (circle, Fig. 2**B**)

A previously healthy 39-year-old female presented with numbness in the left hemiface for two weeks. She denied history of vascular risk factors, malignancy and certain medication. Also, she denied recent history of COVID-19 infection or COVID-19 vaccination before the symptom onset. At first, she had developed multiple vesicles and crusts with oozing, localized to the left side of the upper lip three weeks ago. The skin lesion did not accompany pain and improved without specific treatment after 5 days. However, in a few days, abnormal sensation (i.e., numbness) newly developed on her left hemiface. Her initial sensory symptom covered three divisions of the trigeminal nerve. At the time of consultation to our hospital, sensory disturbance involving the V2 and V3 divisions of the trigeminal nerve was aggravated, whereas numbness in the V1 division was improved. Headache, fever, or neck stiffness were absent on admission. Neurological examination revealed decreased sensation of pain, temperature, and crude touch in the left hemiface involving the V2–V3 divisions of the trigeminal nerve. Corneal reflexes and other cranial nerve evaluations were normal. Other neurological deficits were also absent. Gadolinium-enhanced brain magnetic resonance imaging (MRI) revealed high signal intensities and asymmetrical thickening with enhancement along the cisternal segment of the left trigeminal nerve. (Fig. 1B) Additionally, brain MR angiography showed multifocal stenoses in the M1 segment of the left middle cerebral artery and the cavernous portion of the left internal carotid artery. (Fig. 2A) Facial nerve conduction study and blink test were normal. Routine serologic tests including blood cell count, and inflammatory markers (i.e., C-reactive protein, erythrocyte sedimentation rate) were all within the normal range. The results of serologic testing for tumor markers and autoimmune markers including rheumatoid factor, anti-nuclear antibody, anti-neutrophil cytoplasmic antibody was negative. Moreover, symptoms and signs suggesting systemic vasculitis were absent. Cerebrospinal fluid (CSF) analysis showed mild pleocytosis (22 cells/µL) with a normal protein level (35.2 mg/dL) and glucose ratio (0.7). However, CSF polymerase chain reaction (PCR) assay for specific anti-viral antibodies including herpes simplex virus (HSV), varicella zoster virus, and human immunodeficiency virus was negative. CSF culture also did not identify a specific pathogen. CSF oligoclonal band was negative and CSF/serum IgG ratio was within the normal range. CSF cytology did not reveal malignant cells.

A tentative diagnosis of trigeminal neuritis as a complication of HL involving the CNS was made considering the clinical, neuroradiological, and laboratory findings of the patient. Therefore, intravenous acyclovir (10 mg/kg every 8 h) was administered for 10 days associated with intravenous methylprednisolone (1000 mg/day) for 5 consecutive days. Symptoms improved rapidly during the period of steroid pulse treatment and intravenous acyclovir administration. At the time of discharge, slight paresthesia remained, but it gradually improved, and at a follow-up 1 month later, it was clearly improved. Brain MRI at the 3-month follow-up demonstrated resolution of previously identified high signal intensity lesions with enhancement and multifocal intracerebral arterial stenoses. (Fig. 1D and Fig. 2B).

## Discussion and conclusion

Our patient had a preceding typical HL infection with vesicles and oozing, localized at the vermilion border of the facial lip one week before the sensory symptom onset. Complications of HL are usually limited to skin lesions [4]. Neurological complications of HL such as cranial neuropathy, cluster headache, and psychosis are possible, but rarely occur [4]. In a literature review, only a few cases of cranial neuropathy associated with HL have been reported, even there were no reports of CNS vasculitis due to invasion of HL [5–7]. Umehara et al. (2012) reported a patient who developed trigeminal neuropathy after typical HL infection despite the negative result of CSF HSV PCR, which was similar to our case [5]. The author suggested that trans-axonal spread of virus along the trigeminal nerve to the spinal trigeminal nucleus and tract (without viral transmission via the CSF) might be associated with the negative test results for CSF HSV PCR [5]. However, the exact mechanism of viral spread into the CNS in HL is still to be elucidated, and spreading through hematogenous or CSF pathways also should be considered.

HL is the most common clinical manifestation of recurrent HSV infection and is usually caused by HSV-1 [4]. Thus, to suspect neurological complications of HL, detection of HSV with PCR in the CSF is considered the diagnostic gold standard with high sensitivity and specificity [8]. However, there were some evidences that a false negative CSF HSV PCR could be recognized, especially in the early stages of disease [8, 9]. Even several previous cases of HSV-1 encephalitis with twice negative results for CSF HSV-1 PCR have been reported [8]. Therefore, the negative result of CSF HSV PCR should be interpretated with cuation and does not entirely exclude the CNS invasion of HSV, especially when there is high clinical suspicion for disease [8, 9]. A previous study found various time intervals (12–27 days) between the onset of facial paralysis in Ramsay Hunt syndrome and the detection of brainstem involvement on brain MRI [10]. Moreover, it has been reported that patients experience worsening or progressive neurological deficits several weeks after the onset of facial paralysis [6]. Indeed, our patient developed sensory disturbance one week after the onset of HL. The temporal evolution of the symptoms and signs, and the considerable time interval between the onset of HL and trigeminal neuritis is probably indicative of viral spread into the CNS after reactivation, and these might affect to the negative result of CSF HSV-1 PCR in our patient.

Fortunately, we made a diagnosis of asymptomatic intracerebral arterial stenoses before more serious complications such as ischemic stroke occur. Given the absence of specific symptoms or serologic autoimmune markers indicating systemic vasculitis, mild CSF pleocytosis, and favorable outcome of anti-viral and anti-inflammatory treatment, it seems more reasonable that intracerebral arterial stenoses in our patient might be derived from vasculopathic change due to HL complicated by CNS invasion, rather than atherosclerosis or a manifestation of systemic vasculitis. However, we still could not exclude a coincidence between the two very distinct pathophysiological processes (i.e., a neuritis together with a large/medium size vasculitis) since we have no confirmation of HSV-1 in the CSF. Moreover, progressive headache as well as CSF pleocytosis are common in CNS vasculitis, the clinical presentations of our patient are thought to be atypical.

Our case has some limitations. HSV has not been definitely confirmed, it can be thought that trigeminal affection, CSF pleocytosis, and cerebral arterial stenosis by HSV are speculate. In addition, vascular stenosis was observed on MR angiography clearly, but digital subtraction angiography, the golden standard diagnostic tool of vessel stenosis, was not performed. Nevertheless, the aforementioned clinical evidence suggests that HSV is possible cause.

HL is usually a self-limiting, benign disease without complications, and isolated hemifacial numbness can be easily overlooked without other combined neurological deficits. However, HL rarely presents as trigeminal neuritis due to CNS involvement that may lead to poor prognosis without early recognition and prompt treatment. Therefore, meticulous evaluation may be necessary if trigeminal neuritis or CNS involving symptoms occur after HL.

## Data Availability

All data and material supporting our study are contained within the manuscript. The data are available from the corresponding author upon reasonable request.

## Abbreviations

CNS: Central nervous system
HL: Herpes labialis
MRI: Magnetic resonance imaging
CSF: Cerebrospinal fluid
HSV: Herpes simplex virus
